# Trends in the prevalence of thrombocytopenia among individuals iInfected with hepatitis C Virus in the United States, 1999-2008

**DOI:** 10.1186/1756-0500-5-142

**Published:** 2012-03-13

**Authors:** Teresa L Kauf, David R Nelson, Jonathan Schelfhout, Joseph AC Delaney, Peter Feng Wang

**Affiliations:** 1 Department of Pharmaceutical Outcomes and Policy, College of Pharmacy, University of Florida, Gainesville, Florida, USA; 2 Department Division of Gastroenterology, Hepatology & Nutrition and Clinical and Translational Science Institute, College of Medicine, University of Florida, Gainesville, Florida, USA; 3 Global Health Economics and Outcomes Research, Bristol-Myers Squibb Company, New York, New York, USA

**Keywords:** Thrombocytopenia, Hepatitis C, Platelets, NHANES

## Abstract

**Background:**

Thrombocytopenia is associated with the natural history of hepatitis C virus (HCV) infection and anti-viral therapy. Recent, national estimates of the clinical burden of thrombocytopenia among HCV-infected individuals in the United States are unavailable. Bi-yearly data from the 1999-2000 to 2007-2008 National Health and Nutrition Examination Surveys (NHANES) were used to examine the prevalence of thrombocytopenia among HCV-infected individuals in the United States.

**Results:**

Among 467 HCV-infected individuals in the survey (weighted population = 3,597,039), mean weighted age was 46.7 years (standard deviation = 15.5) and 61.7% were male. Overall, 7.6% met the study definition of TCP at the 150 × 10^9^/L threshold; 4.5%, 2.0%, and 0.8% had platelet counts below 125, 100, and 75 × 10^9^/L, respectively. The 2-year weighted prevalences of thrombocytopenia (150 × 10^9^/L threshold) from 1999-2008 were 4.9%, 8.6%, 6.5%, 4.1%, and 12.9%. The unadjusted biannual time trend (odds ratio) was 1.16 (95% confidence interval = 0.82-1.64). In the two adjusted models, the odds by time ranged from 1.24-1.40, depending on whether the model included demographic or laboratory variables or both, but did not reach statistical significance. Age was positively and significantly related to thrombocytopenia status.

**Conclusions:**

As the HCV-infected population ages, the prevalence of thrombocytopenia is expected to rise. This study provides limited evidence of such an effect at the national level.

## Background

Thrombocytopenia, which may be defined generally as a platelet count below 150 × 10^9^/L, is a common complication of chronic hepatitis C virus (HCV) infection, particularly among those with advanced liver disease, and those receiving antiviral treatment for HCV [1-4]. Estimates of the prevalence of thrombocytopenia in liver disease vary widely, depending on the population and the platelet threshold used [5,6]. Among studies of chronic liver disease patients where thrombocytopenia was defined as a platelet count less than 150 × 10^9^/L, prevalence estimates range from 16-45% [6]. In clinical trials of interferon-based therapy, more than one third of patients experienced a decrease in platelet counts, with counts falling below 50 × 10^9^/L observed in 4-8% of patients [7,8]. Thrombocytopenia while on antiviral therapy may lead to dose reductions, interruption, or discontinuations of interferon, potentially jeopardizing the chances of achieving sustained virologic response (SVR). In cases of portal hypertension and severe thrombocytopenia, HCV patients may be unable to initiate antiviral treatment due to low platelets.

An understanding of trends in the prevalence of HCV and its complications facilitates public health planning and treatment efforts. Although the current incidence of HCV infection is low, the aging of individuals infected with HCV over the past several decades suggests that the burden of liver disease in the United States (US) will continue to increase, with a peak in cirrhosis cases in the year 2020 preceding peaks in liver cancer in the following decade [9]. As that happens, the prevalence of thrombocytopenia within the HCV-infected population also is likely to increase. However, recent projections of HCV and liver disease burden in the US have not considered the prevalence of thrombocytopenia [9-12]. Similarly, available estimates of the future cost of HCV which are based on past treatment patterns may not reflect adequately the additional cost associated with an increased prevalence of thrombocytopenia.

The most recent examination of thrombocytopenia at the national level was conducted using the National Health and Nutrition Examination Survey (NHANES) III [13]. In that study, 13% of individuals with positive HCV antibody had platelet counts below 175 × 10^9^/L. While NHANES III spans the period 1988-1994, it is not possible to determine trends across that time period due to the nature of data collection during that survey phase. Newer administrations of NHANES, however, do permit an examination of trends in the US over consecutive, two-year periods. The objective of this study was to document national trends over the past decade in the prevalence of thrombocytopenia among individuals infected with HCV, with the goal of informing efforts to model the prevalence of complications associated with chronic HCV infection.

## Results

Across the 10-year study period, 467 NHANES participants were identified as having confirmed HCV infection; platelet counts were available for 465 (99.6%). The raw HCV frequency translates to an estimated prevalence in the United States of 3,597,039 individuals (1.48% prevalence), including both diagnosed and undiagnosed cases of infection. Weighted summary characteristics for HCV-infected participants are shown in Table 1. Mean weighted age was 46.7 years and 61.7% were male. Table 1 also summarizes HCV-infected individuals with and without thrombocytopenia (defined as platelet count below 150 × 10^9^/L). Compared to those with normal platelet counts, individuals with low platelet tended to be older (55.3 vs 46.0 years) and have higher ALT (81.5 vs 50.0), AST (84.6 vs 44.2), and iron (138.9 vs 106.9) levels, respectively (p < 0.05 for all comparisons). Questionnaire items tend to have a large number of missing values, and a limited number of participants were eligible for certain examinations, such as fasting glucose and fasting insulin; these items should be interpreted with caution.

**Table 1:** Confirmed HCV infection study sample characteristics

Characteristic	Confirmed HCV-Infected Raw Frequency (n = 467)	Confirmed HCV-Infected Weighted Mean (SD) or Proportion		
		All HCV Patients	Thrombocytopenia Population^1^ (n = 43)	Non-Thrombocytopenia Population^1^ (n = 422)
Age at screening (yrs), mean (SD)	467	46.7 (15.5)	55.3 (22.5)	46.0 (12.7)
Male gender, %	293	61.7	73.4	61.2
Weight (kg), mean (SD)	451	78.2 (22.5)	78.0 (23.3)	78.3 (22.6)
Race/ethnicity, %	467			
White	190	65.7	21.6	20.0
Black/African-American	160	20.1	56.3	66.4
Hispanic	103	10.9	19.3	10.3
Other	14	3.3	2.9	3.3
Annual Household income, %	433			
Below $20,000	185	36.3	39.5	35.6
$20,000 - $74,999	204	50.1	47.1	50.7
Above $75,000	44	13.6	13.4	13.7
Ever used marijuana or hashish, %^1^	124	87.4	100.0	88.0
Times received health care over past 12 months, %	467		43	422
None	83	16.5	13.0	16.9
1-3 times	162	35.6	39.7	34.9
More than 4 times	222	47.9	47.3	48.3
Alcohol use (drinks/day over past year), mean (SD)	270	4.4 (3.7)	4.1 (2.9)	4.4 (3.7)
Albumin (g/dL), mean (SD)	456	4.2 (0.4)	3.7 (1.01)	4.2 (0.4)
ALT (u/L), mean (SD)	456	52.2 (60.9)	81.5 (93.6)	50.0 (56.5)
AST (u/L), mean (SD)	456	47.2 (49.7)	84.6 (46.7)	44.2 (48.5)
Blood urea nitrogen (mg/dL), mean (SD)	456	12.1 (6.4)	13.9 (11.1)	12.0 (5.3)
Total calcium (mg/dL), mean (SD)	456	9.4 (0.5)	9.1 (0.7)	9.5 (0.5)
Creatinine (mg/dL), mean (SD)	346	0.9 (0.3)	1.0 (0.6)	0.9 (0.3)
Iron (ug/dL), mean (SD)	456	108.9 (58.0)	138.9 (61.7)	106.9 (57.2)
Bilirubin (umol/L), mean (SD)	456	13.1 (7.6)	19.3 (13.1)	12.6 (6.9)
Total protein (g/dL), mean (SD)	456	7.4 (0.8)	7.3 (0.9)	7.4 (0.9)
Globulin (g/dL), mean (SD)	456	3.3 (0.8)	3.6 (0.7)	3.2 (0.8)
Hemoglobin (g/dL), mean (SD)	465	14.9 (2.1)	14.5 (2.2)	14.9 (2.1)
Fasting glucose (mg/dL), mean (SD)^2^	91	107.7 (38.9)	105.6 (21.2)	108.0 (42.8)
Fasting insulin (uU/mL), mean (SD)^2^	86	13.3 (12.3)	26.4 (33.9)	11.5 (12.7)
White blood cell count (1000 cells/uL), mean (SD)	465	7.3 (3.2)	5.4 (2.3)	7.4 (3.2)
Platelet count	465	257.6 (87.7)	112.0 (31.2)	269.5 (76.0)
Thrombocytopenia, %	465			
< 150 × 10^9^/L	43	7.6	100	0
< 125 × 10^9^/L	25	4.5	59.4	0
< 100 × 10^9^/L	12	2.0	26.7	0
< 75 × 10^9^/L	7	0.8	10.7	0

HCV = hepatitis C virus; SD = standard deviation; ALT = alanine aminotransferase; AST = asparate aminotransferase

NOTES:

1. Defined as platelet count < 150 × 10^9^/L

2. Among participants 20 years of age and older for survey years 2005-2006 and 2007-2008

3. Among participants 12 years of age and older who attended a morning examination session for survey years 2005-2006 and 2007-2008

**Figure 1:**
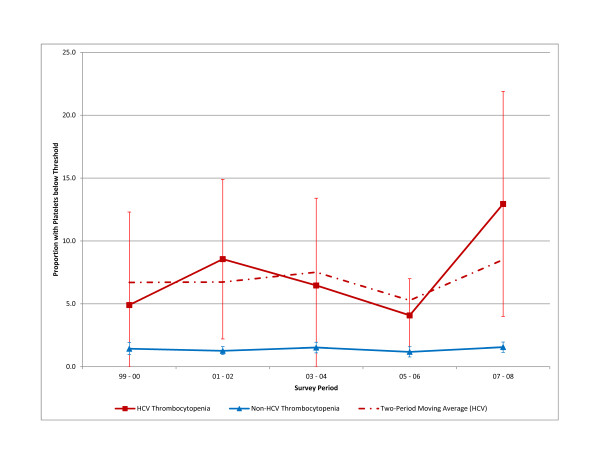
**Prevalence of platelet counts below 150 × 10^9^/L among individuals with confirmed HCV infection and all NHANES participants, 1999-2008**. Note: Error bars represent 95% confidence interval for the proportion of HCV patients with TCP. HCV = hepatitis C virus; NHANES = National Health and Nutrition Examination Survey; TCP = thrombocytopenia.

Figure 1 shows the 2-year point estimates and 95% confidence intervals for the prevalence of thrombocytopenia at the 150 × 10^9^/L threshold from 1999-2008 for participants with and without confirmed HCV infection. Trends for lower thresholds were unstable due to very small sample sizes and are not shown. Among HCV-infected individuals, the prevalence of thrombocytopenia at the 150 × 10^9^/L threshold increased from 4.9% in 1999-2000 to 12.9% in 2007-2008, but first increased then decreased from 2001-2006, although none of the periods showed a statistically significant change from other periods. Trends in thrombocytopenia prevalence among non-HCV-infected individuals followed a similar pattern, although at much lower levels. The rise in thrombocytopenia prevalence of 8 percentage points among those with confirmed HCV infection represented a 164.0% increase but did not reach statistical significance. In contrast, thrombocytopenia prevalence among non-HCV-infected individuals was relatively stable during the same time period, increasing by less than 9%, from 1.4% to 1.6%.

Platelet counts among patients with HCV were significantly correlated with several laboratory measures (data not shown but available from the authors by request). Those not correlated with platelets included protein, fasting glucose, and fasting insulin, although the ability to detect relationships to the fasting variables was hampered by limited data availability. ALT and AST were significantly correlated with bilirubin, protein, hemoglobin, iron, and globulin. Of these, iron, protein, and globulin were excluded from further analysis because they showed particularly strong correlations with ALT and AST.

Results for the three time-trend regression models are shown in Table 2. The univariate, biannual time trend (odds ratio) for platelets less than 150 × 10^9^/L was 1.16 (95% CI = 0.82-1.64). The point estimate, which did not reach statistical significance, can be interpreted as a bi-yearly increase in thrombocytopenia prevalence of 16%. In adjusted models, the odds by time increased to 1.24-1.40, depending on whether the model included demographic or laboratory variables or both, but failed to reach statistical significance. Age was positively associated with an increase in the odds of thrombocytopenia in both adjusted models. None of the laboratory measures was significantly related to thrombocytopenia status, although the coefficients were of the expected sign. The presence of thrombocytopenia was not associated with ALT or AST level.

**Table 2:** Adjusted time-trend regression results

	HCV-Confirmed Patients (n = 467)		
Variable	Model 1 n = 465 OR (95% CI)	Model 2 n = 449 OR (95% CI)	Model 3 n = 440 OR (95% CI)
Time	1.16 (0.82-1.64)	1.24 (0.84-1.84)	1.40 (0.88-2.22)
Age (years)		1.05 (1.02-1.08)	1.02 (1.02-1.12)
Male gender		2.87 (0.97-8.51)	1.45 (0.30-6.94)
Weight (kg)		0.99 (0.96-1.02)	0.99 (0.95-1.04)
Race/ethnicity (vs White)			
Black/African-American		1.26 (0.47-3.37)	0.50 (0.10-2.52)
Hispanic		1.19 (0.38-3.72)	0.94 (0.27-3.30)
Other		1.18 (0.13-11.06)	1.09 (0.17-7.00)
Albumin			0.28 (0.04-2.21)
ALT			1.01 (0.99-1.03)
AST			1.01 (0.99-1.03)
Blood urea nitrogen			0.92 (0.84-1.01)
Total calcium			0.18 (0.02-2.23)
Bilirubin			1.05 (0.99-1.13)
Hemoglobin			1.07 (0.73-1.56)
White blood cell count			0.59 (0.73-1.56)
Model c-statistic	0.48	0.74	0.89

HCV = hepatitis C virus; OR = odds ratio; CI = confidence interval; ALT = alanine aminotransferase; AST = asparate aminotransferase

## Discussion

This study is the first to report trends in the prevalence of thrombocytopenia across a national sample of individuals infected with HCV. For the most recent time period available, 2007-2008, the prevalence of platelets below 150 × 10^9^/L among HCV patients reached nearly 13%. Further, the prevalence over time among the non-HCV-infected US population remained fairly stable. While there was a dip in thrombocytopenia prevalence for survey years 2005-2006, the presence of a similar dip among the weighted population of all NHANES participants suggests that the decrease could be a function of laboratory processing or some other systematic change during that survey round.

A prior study utilizing NHANES III data for the years 1988-1994 found similar thrombocytopenia prevalence among HCV-infected individuals. In that study, however, a higher platelet threshold of 175 × 10^9^/L-the bottom 5% of platelet counts across all participants-was used [13]. If platelet counts remained stable, then the prevalence estimates from the current study, at a lower threshold, should have been lower than in 1988-1994. We also found age to be positively and significantly related to thrombocytopenia status. Taken together, these two studies suggest that the prevalence of thrombocytopenia among individuals infected with HCV likely increased-perhaps substantially-over the past two decades. However, the spike in thrombocytopenia prevalence among HCV-infected individuals observed in 2007-2008 could be an outlier. Additional years of data will be needed to delineate a clear trend.

Several previous studies have examined platelet counts among HCV patient populations. Louie et al (2011) performed a systematic review of those studies conducted in populations of 50 or more confirmed HCV-infected patients. Among the 9 studies which used a platelet threshold of 150 × 10^9^/L to define thrombocytopenia, reported prevalence ranged from 16.0% to 41.0% [6]. The much higher proportions reported in these studies compared to the current analysis may be explained, in part, by the inclusion in the present study of individuals with undiagnosed HCV infection. Davis et al (2010) estimates that approximately 70% of individuals infected with HCV are unaware of their serostatus [9]. Among the 167 NHANES participants from 1999-2008 with confirmed HCV infection who responded to a follow-up telephone survey, 48.9% reported that they were previously unaware of their serostatus. It is likely that a large proportion of those patients have yet to exhibit symptoms of liver disease, and one would expect a lower prevalence of thrombocytopenia among such patients.

The use of NHANES presents both advantages and disadvantages for the examination of thrombocytopenia among individuals infected with HCV. The primary advantage of NHANES lies in its sampling strategy, which allows for the examination of a nationally representative population of individuals. Additionally, NHANES has been conducted on a biennial basis since 1999, allowing for the examination of disease trends over the past decade. The present study examines two conditions of which patients may be unaware. HCV infection may remain asymptomatic for several decades, such that many individuals infected with HCV are unaware of their status [9]. Similarly, thrombocytopenia is a laboratory abnormality that may not appear as a specific diagnosis in administrative data and patients may or may not identify it as a (known) medical condition. Claims data or self-reported surveys thus would be inadequate to examine the prevalence of thrombocytopenia within the HCV-infected population.

NHANES does present some limitations. Since HCV is relatively uncommon among the general population, sample sizes within each 2-year survey period are small, limiting the ability to detect changes over time, particularly for lower platelet counts which may be more relevant to clinical practice. The small sample also contributes to the volatility in the estimates; a difference of just one or two cases could have a large impact on the prevalence estimates. Further, some high-risk groups such as the homeless and incarcerated individuals are not represented. Because those groups have not been adequately studied, it is not known whether the risk of thrombocytopenia with such risk groups is higher or lower than among the overall HCV patient population. Finally, NHANES is a cross-sectional survey with limited information about patients' medical histories, including duration of HCV infection, liver disease status, treatment history, and mode of transmission. Because NHANES lacks this information, it is not possible to model the relationship between chronic liver disease and thrombocytopenia. In particular, duration of HCV infection is an unobserved latent variable that may be explaining part of the variation in thrombocytopenia rates.

Davis et al (2010) predict that the prevalence of cirrhosis will peak in 2020 [9]. Previous investigators have suggested that the future burden of liver disease is likely to be reduced only through improvements in the effectiveness of antiviral therapy and increases in the numbers of patients receiving therapy [9-12,14,15]. The latter goal could be positively affected through the use of emerging treatments which may safely improve platelet counts among HCV-infected patients [5,16]. One study has shown that treatment for low platelets can improve the ability of some patients to initiate antiviral treatment [17], but further study is needed to see whether the use of such therapies will help patients initiate, maintain, and complete antiviral treatment. Moreover, non-peginterferon-based treatment strategies for chronic HCV infection, several of which are under investigation, may enable more patients to initiate and complete anti-viral therapy and have a higher likelihood of SVR [18].

## Conclusions

Thrombocytopenia increases health costs [5,19] and may interfere with the ability of some patients to initiate or tolerate antiviral therapy [1,2,17]. If the burden of HCV is to be reduced, the clinical impact of thrombocytopenia in HCV-related liver disease must be addressed. A substantial proportion of HCV patients have low platelet counts, and the prevalence of low platelets among individuals with chronic liver disease is likely to increase through the coming decade. Future efforts to estimate the human and economic consequences of HCV infection should consider the role of thrombocytopenia as both a clinical manifestation of disease and a possible barrier to treatment.

## Methods

### Data and patients

Multiple years of NHANES data were used to examine changes in the prevalence of thrombocytopenia among HCV patients in the US from 1999-2008 [20]. NHANES consists of an interview and comprehensive medical examination of a nationally representative sample of about 5,000 persons each year. The interview component includes demographic, socioeconomic, dietary, and health-related questions. The examination component consists of medical, dental, and physiological measurements, as well as laboratory tests administered by highly trained medical personnel. NHANES study participants aged 6 years and older were eligible for hepatitis C antibody testing, which was repeated in duplicate for positive samples.

All participants with available information on hepatitis C testing and evidence of confirmed infection (positive Chiron RIBA 3.0 Strip Immunoblot Assay) were eligible for inclusion in the current study [20]. NHANES participants also received a complete blood count with differential in whole blood, which included platelet count and mean platelet volume, and a standard biochemistry profile. Included in the latter were alanine aminotransferase (ALT) and asparate aminotransferase (AST). Self-reported information about prescription drug use during the past 30 days and medical conditions were included in the questionnaire portion of the survey.

### Study measures

The primary measure of interest for the study was thrombocytopenia. Thrombocytopenia was defined as platelet count below 150 × 10^9^/L, although other thresholds were examined. Laboratory and examination variables used to assess the likelihood of thrombocytopenia included ALT, AST, iron, fasting glucose, fasting insulin, albumin, creatinine, bilirubin, total protein, hemoglobin, white blood cell count, and weight. Questionnaire variables included alcohol use, marijuana use, health care utilization over the past 12 months, and prior diagnosis of liver cancer. Demographic variables included age, gender, race/ethnicity, and income.

### Statistical analysis

Summary statistics for the variables noted above were calculated for all confirmed HCV-infected NHANES participants. The proportion of confirmed HCV-infected participants meeting a threshold criterion for thrombocytopenia was calculated for each 2-year survey period and compared using Fisher's exact test. The proportion of all NHANES participants with low platelets in each period was provided as a comparison.

Trends over time were analyzed via a series of logistic regression models to account for non-linear relationships between variables. The dependent variable in each model was a dichotomous marker of thrombocytopenia (yes/no) for each subject. The initial model considered only time, with each 2-year survey timeframe considered as one period. Subsequent models included time plus demographic variables (Model 2) and time plus demographic variables plus laboratory measures (Model 3). Pearson correlation coefficients between laboratory measures were calculated. Measures not significantly correlated with platelet counts were excluded from the regression analysis to arrive at a more parsimonious model. Similarly, laboratory measures which were significantly and strongly correlated with ALT and AST were excluded to avoid problems with multi-colinearity and improve model fit. Strong correlations were defined as correlation coefficients with absolute values exceeding 0.20. Questionnaire items with low response rates were not included due to missing data. Odds ratios and 95% confidence intervals were reported for each model.

Summary statistics, prevalence estimates, and regression analyses were weighted to represent the US population using survey weights provided by the National Center for Health Statistics. All analyses were conducted using SAS version 9.2, with 2-sided tests of hypothesis. P-values of 0.05 or lower were considered statistically significant. The study was approved by the University of Florida Institutional Review Board. All authors read and approved the final manuscript.

## Abbreviations

HCV: hepatitis C virus; SVR: sustained virologic response; US: United States; NHANES: National Health and Nutrition Examination Survey; ALT: alanine aminotransferase; AST: asparate aminotransferase.

## Competing interests

TLK has received funding from the study sponsor, GlaxoSmithKline, Inc, for this and other studies. DRN has served as a consultant for GlaxoSmithKline, Inc. PFW was a full-time employee of GlaxoSmithKline, Inc. at the time of the study. JACD and JS have no conflicts to disclose.

## Authors' contributions

This study was conceived by TLK and DRN and executed by TLK, JS, and JACD. PFW provided important background information and served as a consultant regarding data analysis and interpretation. TLK drafted the manuscript, and all authors provided important intellectual contributions to the content. Full responsibility for all results and conclusions lies with the corresponding author.
